# Bone marrow stromal cells reduce low-dose cytarabine-induced differentiation of acute myeloid leukemia

**DOI:** 10.3389/fphar.2023.1258151

**Published:** 2023-10-26

**Authors:** Tomislav Smoljo, Barbara Tomic, Hrvoje Lalic, Vilma Dembitz, Josip Batinic, Antonio Bedalov, Dora Visnjic

**Affiliations:** ^1^ Laboratory for Cell Biology, Department of Physiology, Croatian Institute for Brain Research, University of Zagreb School of Medicine, Zagreb, Croatia; ^2^ Division of Hematology, Department of Internal Medicine, University Hospital Centre Zagreb, Zagreb, Croatia; ^3^ Clinical Research Division, Fred Hutchinson Cancer Research Centre, Seattle, WA, United States

**Keywords:** cytarabine, differentiation, acute myeloid leukemia, bone marrow stromal cells, cell cycle

## Abstract

Low-dose cytarabine (LDAC) is a standard therapy for elderly acute myeloid leukemia (AML) patients unfit for intensive chemotherapy. While high doses of cytarabine induce cytotoxicity, the precise mechanism of action of LDAC in AML remains elusive. *In vitro* studies have demonstrated LDAC-induced differentiation; however, such differentiation is seldom observed *in vivo*. We hypothesize that this discrepancy may be attributed to the influence of bone marrow (BM) stromal cells on AML cells. Thus, this study aimed to investigate the impact of BM stromal cells on LDAC-induced differentiation of AML cell lines and primary samples. Our results demonstrate that the presence of MS-5 stromal cells prevented LDAC-induced cell cycle arrest, DNA damage signaling and differentiation of U937 and MOLM-13 cell lines. Although transcriptomic analysis revealed that the stroma reduces the expression of genes involved in cytokine signaling and oxidative stress, data obtained with pharmacological inhibitors and neutralizing antibodies did not support the role for CXCL12, TGF-β1 or reactive oxygen species. The presence of stromal cells reduces LDAC-induced differentiation in primary samples from AML-M4 and myelodysplastic syndrome/AML patients. In conclusion, our study demonstrates that BM stroma reduces differentiation of AML induced by LDAC. These findings provide insights into the limited occurrence of terminal differentiation observed in AML patients, and suggest a potential explanation for this observation.

## 1 Introduction

Acute myeloid leukemia (AML) is a heterogeneous group of hematological malignancies with high relapse rates and poor overall survival. AML is characterized by impaired differentiation and uncontrolled clonal expansion of myeloid precursors, making therapy aimed at differentiation a promising treatment strategy. The most successful example of differentiation therapy is all-*trans* retinoic acid (ATRA)-based treatment of acute promyelocytic leukemia (APL), which turned once fatal disease into the most curable subtype of AML. Although ATRA differentiated non-APL AML cell lines *in vitro* and showed some activity in primary samples *ex vivo*, these findings did not translate into clear clinical benefit for other subtypes of AML (Madan and Koeffler, 2020). Recently approved targeted therapies for AML subtypes carrying mutations of isocytrate dehydrogenase (*IDH*) and activating mutations of *FLT3* revived interest in differentiation therapy. Ivosidenib and enasidenib, inhibitors of mutated IDH1 and IDH2, induced myeloid differentiation and durable remissions in patients with multiple comorbidities (Amatangelo et al., 2017; Stein et al., 2017; Roboz et al., 2020). Gilteritinib, a type I FLT3 inhibitor, stimulated differentiation towards granulocytic and monocytic lineage in about half of the *FLT3*-mutant patients (McMahon et al., 2019), and quizartinib, a type II FLT3 inhibitor, caused terminal myeloid differentiation of leukemic blasts with a surge in the number of *FLT3*-mutated neutrophils (Sexauer et al., 2012). An increasing number of drugs, including inhibitors of histone deacetylase, pyrimidine synthesis inhibitors or cell cycle regulators, have been shown to stimulate differentiation of leukemic blasts in preclinical models, but their single-agent activity in clinical trials has not been proven (Madan and Koeffler, 2020).

Although not initially developed as new forms of differentiation therapy, many drugs used in the treatment of AML promote differentiation as part of their therapeutic effects. Cytarabine (AraC), a backbone of the standard induction therapy in adults with AML, has been suggested to be capable of triggering AML remissions without toxicity when applied at low doses *in vivo* (de Thé, 2018). At standard dose, AraC is combined with an anthracycline for the treatment of patients with newly diagnosed AML who are suitable candidates for intensive chemotherapy. However, majority of AML patients are older patients who are unfit for intensive chemotherapy so that an acceptable low-intensity therapy includes low-dose cytarabine (LDAC) (Short et al., 2020). Cytotoxic effect of high doses of cytarabine on AML cells is ascribed to apoptosis, but the effects of LDAC remained incompletely understood. In AML cell lines, LDAC induces granulocytic or monocytic differentiation (Wang et al., 2000; Chen et al., 2017) that depends on the activation of DNA damage signaling pathway (Tomic et al., 2022; Wang et al., 2022). However, terminal differentiation in patients treated with LDAC are rarely described so that cytarabine has been used in differentiation therapy mostly to enhance the effects of other differentiation-inducing agents (Madan and Koeffler, 2020).

One of the factors *in vivo* that may contribute to the failure to translate pre-clinical findings to a clinically successful strategy of differentiation therapy is the role of the bone marrow (BM) microenvironment. The protective effect that BM stroma confers on leukemia cells in response to various cytotoxic drugs is well described (Ciciarello et al., 2021). However, the effects of stroma on AML differentiation are less investigated and results depend on agents used. It has been described that co-culture of stromal cells and AML-M2 HL-60 cells *in vitro* reduced ATRA-mediated differentiation (Su et al., 2015), which might help to explain why ATRA has activity against non-APL AML *in vitro*, but limited clinical activity. The opposite effect was observed in the co-culture of primary blasts and human BM stroma, in which FLT3 inhibition with quizartinib induced cell-cycle arrest and differentiation rather than apoptosis. These results parallel the responses observed clinically, in which peripheral blasts are rapidly cleared whereas BM blasts remain viable but undergo differentiation (Sexauer et al., 2012). The protective effect of stromal MS-5 cells on AraC-mediated apoptosis has been described (Konopleva et al., 2002; Moschoi et al., 2016; Di Tullio et al., 2017), but the effects on differentiation induced by LDAC has not been investigated.

In the present study, we tested the effects of the presence of BM stromal cells on differentiation and proliferation of AML cells in response to various doses of AraC. We hypothesize that the limited and infrequent differentiation outcomes observed in LDAC-treated patients could be attributed, in part, to the influence exerted by the bone marrow microenvironment on AML cells *in vivo*.

## 2 Materials and methods

### 2.1 Reagents

Reagents used are listed in Supplementary Table S1.

### 2.2 Cell lines

U937 and MOLM-13 AML cell lines were maintained in RPMI 1640, with 10% fetal bovine serum, 2 mM L-glutamine, penicillin (50 U/mL), and streptomycin (50 μg/mL). Mouse stromal MS-5 cell line was maintained in alpha-MEM (with ribo- and deoxyribonucleosides), with 10% fetal bovine serum, 2 mM L-glutamine, 2 mM sodium pyruvate, penicillin (50 U/mL) and streptomycin (50 μg/mL) at 37 °C in a humidified atmosphere containing 5% CO_2_.

### 2.3 Co-cultures

For the experiments, MS-5 cells were seeded at a starting concentration of 90 × 10^3^/well in 6-well plates or 480 × 10^3^/25 cm^2^ flasks 24 h before the addition of AML cell lines. Co-cultures of AML cell lines with MS-5 confluent monolayer were performed in RPMI 1640. Exponentially growing AML cells were seeded at a starting concentration of 0.2 × 10^6^/mL in 6-well plates or 0.3 × 10^6^/mL in 25 cm^2^ flasks. The agents tested were added at concentrations indicated in the Figure legends. In some experiments, 24 mm transwell cell culture inserts with 0.4 μm pore polyester membrane was used to separate AML cells from MS-5 monolayers.

### 2.4 Intracellular reactive oxygen species (ROS)

Intracellular ROS were detected by staining cells with dihydrorhodamine 123 (DHR123). At the end of incubation, U937 or MS-5 cells were collected in PBS or PBS with 1% bovine serum albumin (BSA) and incubated with or without 100 μM DHR123 at 37 °C in dark conditions for 15 min. Cells were analyzed by flow cytometry using the FACSCanto II or FACSLyric (BD Biosciences, San Jose, CA, United States), and the mean fluorescence intensity (MFI) of the sample was determined by FlowJo v.10 platform.

### 2.5 Cell proliferation, cell cycle and cell death analyses

Viable cells were counted by trypan blue exclusion at the indicated time points. For cell cycle analysis, an aliquot of cells was stained directly with propidium iodide (PI) solution, as previously described (Dembitz et al., 2019), using the Attune acoustic focusing cytometer (Life Technologies, ABI, Carlsbad, CA, United States) or FACSCanto II flow cytometer. Collected data were processed using doublet discrimination and automatic assignment of cell cycle boundaries according to the propidium iodide intensity using the FlowJo cell cycle platform. Apoptosis was measured using an annexin V—fluorescein isothiocyanate (FITC) kit, according to the manufacturer’s instructions. Samples were analyzed for the percentage of annexin V - FITC and propidium iodide (PI)- positive cells using the FACSLyric system and FlowJo v.10 platform.

### 2.6 Morphological analysis

At the end of experiment, 50,000 AML cells per sample were cytospun on microscopic slides (1,000 rpm, 2 min) using StatSpin Cytofuge 2 (BeckmanCoulter, Marseille, France) and left to dry overnight. Adherent stromal cells were washed with PBS and left to dry for at least 2 h. Air-dried slides were stained with May-Grünwald Giemsa stain, as previously described (Tomic et al., 2022). Morphology was examined using AxioVert 200 microscope and images were obtained using AxioCam MRc 5 camera and ZEN software, blue edition (Carl Zeiss AG, Oberkochen, Germany).

### 2.7 Total cell lysates and Western blot

U937 and MOLM-13 cells were plated at the concentration of 0.3 × 10^6^/mL in 25 cm^2^ flasks as indicated in Figure legends. At the end of incubation, total cell lysates were isolated as previously described (Dembitz et al., 2019). Briefly, cells were collected and resuspended in cell lysis buffer containing freshly added 1 mM phenylmethylsulfonyl fluoride and 1 μM microcystin and incubated on ice for 10 min. After incubation, cells were disrupted by seven passages through 23-gauge needle and incubated on ice for additional 10 min. After centrifugation at 14,000 x g at 4 °C for 10 min, supernatants were collected and stored at −80 °C. The protein concentration of each sample was determined colorimetrically using Bradford reagent and Eppendorf Biophotometer Plus at 595 nm.

Equal amounts of protein extracts (50 μg/well) were mixed with LDS Sample Buffer (4X) and run on 4%–12% SDS–polyacrylamide gel and transferred to PVDF membrane using Mini-PROTEAN Tetra electrophoresis system (Bio-Rad Laboratories, Hercules, CA, United States). Anti-Chk1, anti-pChk1 (S345), anti-cell-division cycle 2 (CDC2), anti-pCDC2 (Tyr15), and monoclonal anti-β-actin primary antibodies were employed at the recommended dilutions. After incubation, membranes were washed and incubated in appropriate anti-mouse IgG or anti-rabbit IgG HRP-linked secondary antibodies for 120 min at room temperature. Protein bands were visualized using SuperSignal™ West Pico PLUS chemiluminescent substrate, ChemiDoc™ MP Imaging System and Image Lab™ software (Bio-Rad Laboratories, Hercules, CA, United States).

### 2.8 RNA sequencing

RNA Sequencing was provided by Novogene UK Company Limited (Cambridge, United Kingdom). Messenger RNA was purified using poly-(A) enrichment. After fragmentation, the first strand cDNA was synthesized using random hexamer primers, followed by the second strand cDNA synthesis using either dUTP for directional library or dTTP for non-directional library. After cluster generation, the library preparations were sequenced on an Illumina NovaSeq 6000 platform, and paired-end reads were generated.

Reads were mapped to a reference genome using Hisat2 and Featurecounts was used to count the read numbers mapped of each gene. Differential expression analysis was performed using the DeSeq2 R package and *p*-values were adjusted using the Benjamini and Hochberg methods. The corrected *p*-value of 0.01 and |log2(Fold Change)| of 1 was set as the threshold for significantly differential expression using the DESeq2 R package. Enrichment analysis based on Gene Ontology (GO) pathways (http://www.geneontology.org/) was performed using the clusterProfiler R package.

Generated data is available in the ArrayExpress database under the identifier E-MTAB-13192.

### 2.9 Cytokine assay

MS-5 cells were cultured alone or in the presence of U937 for 72 h. At the end of incubation, media were collected and centrifuged at 1,000 rpm for 5 min, supernatants were collected and stored at −20°C. The levels of IL-34, IL-5, M-CSF, GM-CSF, TGF-β1, SCF, and CXCL12 (stromal cell-derived factor-1, SDF-1) were measured using LEGENDplex™ Mouse HSC Myeloid Panel (7-plex) according to manufacturer’s instructions. Briefly, samples and standards were thawed and incubated with capture beads for 2 h. After incubation, the plate was washed and a biotinylated detection antibody cocktail is added. After 1 h incubation, Streptavidin-phycoerythrin (SA-PE) was added and incubated for 30 min. Samples were acquired on BD FACSCanto II and analyzed using LEGENDplex™ Data Analysis Software (BioLegend, San Diego, CA, United States).

### 2.10 Primary AML samples cell culture

Bone marrow samples were obtained upon written informed consent from two patients with non-APL AML. The study was performed according to the Declaration of Helsinki and approved by the Institutional Review Board of the University of Zagreb School of Medicine (380-59-10106-17-100/94) and University Hospital Centre Zagreb (02/21 AG). In routine diagnostic procedures, the samples were evaluated for the presence of *FLT3* mutations and cytogenetic abnormalities. Samples with normal karyotypes were additionally tested for *NPM1* mutations. Further analyses of *BCR-ABL*, *RUNX1/RUNX1T1*, *PML-RARA*, *CBFB/MYH11*, and *KMT2A-AFF1/MLLT3* were performed depending on the French-American-British (FAB) subtype. AML patients’ characteristics are Pt 07 (FAB M4, normal karyotype 46 XX, *FLT3*-ITD, *NPM1* mutated) and Pt 09 (secondary AML progressed from myelodysplastic syndrome (MDS), normal karyotype 46 XY).

Diagnostic bone marrow aspirates were separated using NycoPrep™ 1.077, as previously described (Dembitz et al., 2022; Tomic et al., 2022). Mononuclear cells from bone marrow samples Pt 07 and Pt 09 were purified by overnight adherence to plastic and then cryopreserved in liquid nitrogen. For the experiments, samples were thawed and resuspended in RPMI 1640 containing 10% FBS, 2 mM L-glutamine, 50 U/mL penicillin, and 50 μg/mL streptomycin. Total cell number and viability were assessed by counting on a hemocytometer using trypan blue exclusion. Viable cells were seeded at the concentration of 0.4 × 10^6^/mL in 12-well plates with or without MS-5 cells, in a medium supplemented with 50 ng/mL interleukin-3 (IL-3), interleukin-6 (IL-6), FLT3 ligand (FLT3L) and stem cell factor (SCF), and treated with agents tested.

### 2.11 Immunophenotyping

At the end of incubation, AML cell lines and primary blasts were stained with appropriate antibodies, as previously described (Dembitz et al., 2020; Tomic et al., 2022). For the analysis of MS-5 cells, nonadherent cells and media were removed, adherent cells were harvested through trypsinization, washed in RPMI, and resuspended in PBS with 1% BSA on ice. Non-viable cells were excluded by 7-AAD staining and forward scatter (FSC) vs. side scatter (SSC) gating, and doublet exclusion was performed by plotting the height or width against the area for FSC. For the analysis of MS-5 cell surface markers, additional CD45 vs. SSC gating was used to exclude adherent human AML cells. Flow cytometry analyses were performed using Attune acoustic focusing cytometer or FACSCanto II flow cytometer, and the obtained data were analyzed by FlowJo v.10 platform. The MFI of the sample was calculated by subtracting the MFI levels of isotypic controls from the MFI levels of the cells stained with CD-specific antibodies. The percentage of positive cells was determined by measuring the fluorescence shift of distinct clusters of leukemic events using the FlowJo v.10 platform.

### 2.12 Statistical analysis

Data are presented as mean ± standard error of the mean (S.E.M). One-way analysis of variance (ANOVA) followed by Tukey’s multiple comparison test was performed using GraphPad Prism version 6.07. The results were considered to be statistically significant if *p* was <0.05.

## 3 Results

### 3.1 The presence of stromal cells prevents cell cycle arrest and differentiation of monocytic U937 cells induced by low-dose AraC

MS-5 stromal cell line, a type of mouse adherent fibroblastic cells growing in monolayers, was previously described to be efficient in supporting leukemic cells (Griessinger et al., 2014). To model the effects of the BM stromal microenvironment on AML *in vitro*, AML cells were cultured in 6-well plates in the presence or absence of MS-5 stromal cell monolayer. Cytarabine was added at concentrations of 10, 100, and 1,000 nM, since previous studies shown that concentrations up to 100 nM induce differentiation *in vitro* and the higher concentrations exerted an increasing cytotoxicity (Wang et al., 2000; Chen et al., 2017). The lower tested concentrations (10 and 100 nM) compare to those observed in plasma of patients receiving 20 mg/m^2^/d by continuous IV as measured at week 1 and week 2 (Spriggs et al., 1985).

**FIGURE 1:**
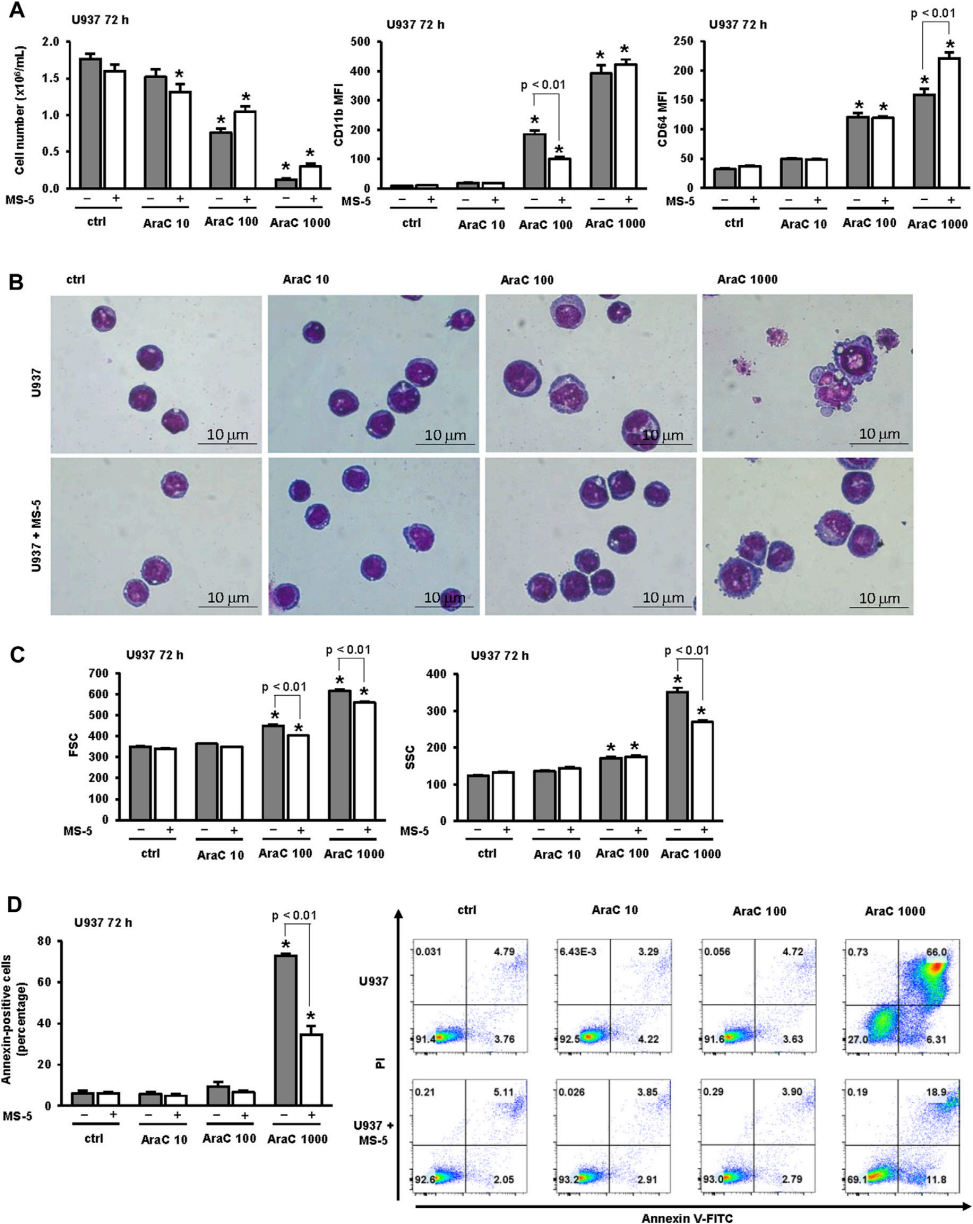
The presence of stromal MS-5 cell line reduces differentiation of U937 cells induced by low-dose AraC. U937 cells were cultured either alone or in the presence of MS-5 stromal cell line and untreated (ctrl) or treated with AraC (10, 100, 1,000 nM) for 72 h. **(A)** The number of viable cells and the expression of differentiation markers. Mean fluorescence intensity (MFI) of CD11b and CD64 was calculated as described under “Materials and methods” section. **(B)** Representative May-Grünwald-Giemsa stained cytospin preparations of U937 cells (×100 magnification). **(C)** Flow cytometry analysis of forward scatter (FSC) and side scatter (SSC) of U937 cells. **(D)** The percentage of annexin-positive U937 cells and the representative dot plots of cells stained with annexin V-FITC/propidium iodide (PI) and analyzed by flow cytometry. Results are mean ± S.E. (error bars) of at least three independent experiments. *, *p* < 0.05 (ANOVA and Tukey) compared with control (ctrl).

When incubated in the absence of stromal cells, U937 cells showed a dose dependent decrease in the number of viable cells (Figure 1A). To test the effects on differentiation, we determined the expression of the marker CD11b, which is most often used as a marker of myeloid differentiation (Wang et al., 2000; Tavor et al., 2008; Chen et al., 2017; Hosseini et al., 2019; McKenzie et al., 2019; Duy et al., 2021; Tomic et al., 2022; Wang et al., 2022), and the expression of CD64, which is a marker of monocytes (Chen et al., 2017; Tomic et al., 2022). LDAC induced an increase in the expression of CD11b and CD64, as previously described (Tomic et al., 2022). The presence of MS-5 monolayer reduced AraC-induced cytotoxicity and decreased the expression of CD11b in cells treated with a lower dose of AraC, while the level of CD11b was high in cells surviving after the treatment with the highest dose of AraC (Figure 1A). Morphological analysis revealed that the presence of stromal cells prevented a decrease in nucleo/cytoplasmic ratio induced by AraC (Figure 1B). Furthermore, the inhibitory effect of the stroma on an increase in cell size and vacuolization was confirmed by flow cytometry analysis (Figure 1C).

Our previous study revealed that the effects of low-dose AraC (100 nM) on the number of viable cells and the expression of CD11b were not prevented by the presence of pan-caspase inhibitor, suggesting that cell cycle arrest in response to low-dose AraC was due to differentiation and not to the loss of viability due to cell death (Tomic et al., 2022). To further dissect the effect of stroma on apoptosis induced by several doses of AraC, the percentage of annexin-positive cells in the presence or absence of stromal MS-5 cell line was compared (Figure 1D). As previously described, the presence of low-dose (100 nM) AraC had no significant effects on the percentage of annexin-positive U937 cells, while the percentage of annexin-positive cells treated with 1,000 nM AraC was significantly increased. The presence of stroma had no effects on apoptosis in cells treated with low-dose AraC, but prevented the increase induced by high dose AraC (1,000 nM). These results suggest that AraC-initiated differentiation precedes an increase in apoptosis and cell death.

**FIGURE 2:**
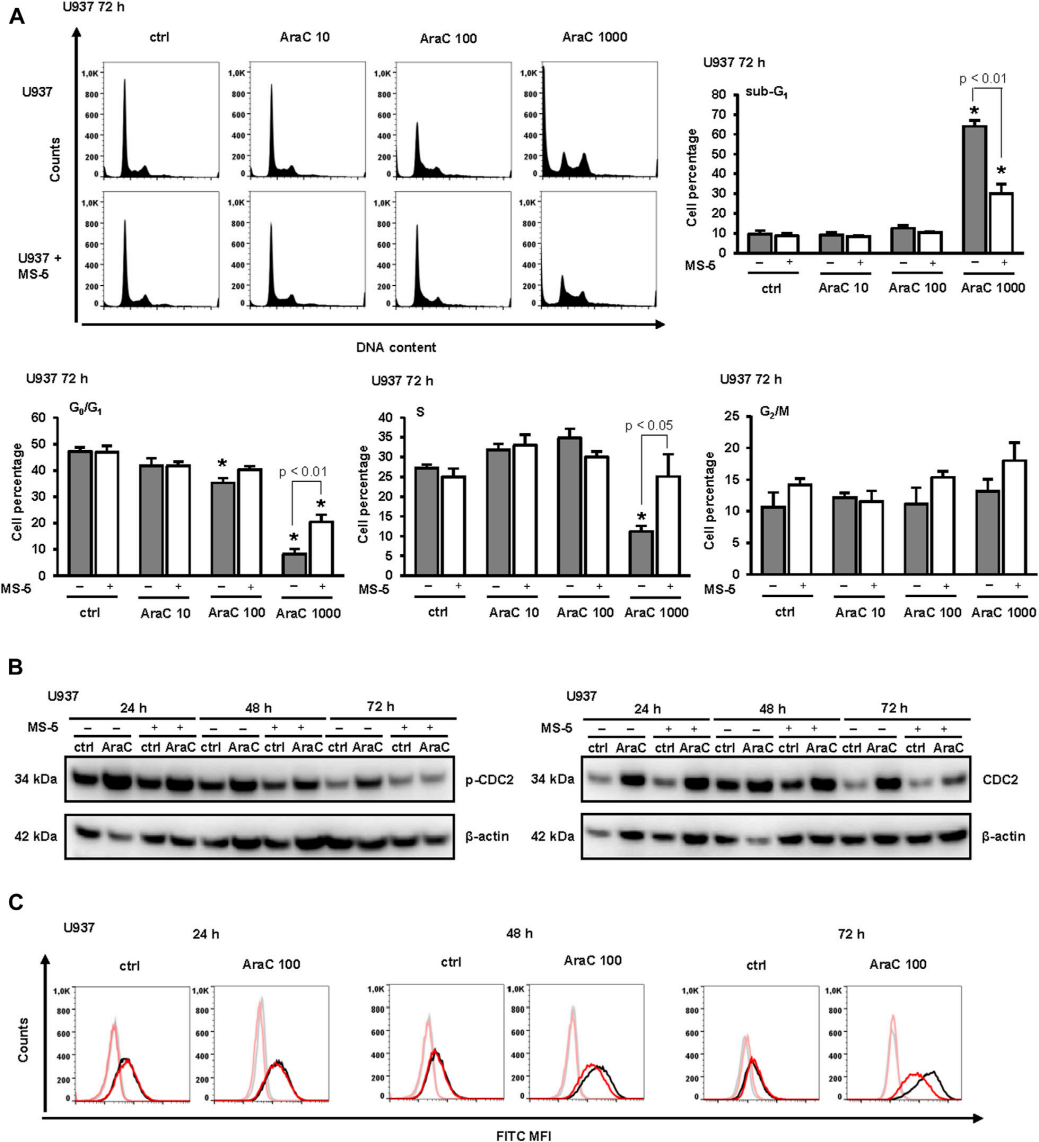
The presence of stromal MS-5 cell line prevented cell cycle arrest of U937 cells and decreased an inhibitory phosphorylation of cell division cycle protein 2 (CDC2)/cyclin-dependent kinase 1 (CDK1). U937 cells were cultured either alone or in the presence of MS-5 stromal cell line and untreated (ctrl) or treated with AraC (10, 100, 1,000 nM). **(A)** The cell cycle progression was analyzed by flow cytometry 72 h after addition of AraC. Representative histograms from propidium-labeled cells and the percentage of cells in sub-G_1_, G_0_/G_1_, S, and G_2_/M phase of the cell cycle. Results are mean ± S.E. (error bars) of at least three independent experiments. *, *p* < 0.05 (ANOVA and Tukey) compared with control (ctrl). **(B)** Total cell lysates of U937 cells were isolated after 24, 48 and 72 h and analyzed by Western blotting for the level of Tyr-15-phosphorylated CDC2 and total CDC2. Representative immunoblots from three independent experiments are shown. **(C)** Aliquots of cells shown in B were analyzed for the expression of CD11b by flow cytometry. Representative histograms with black line representing the expression of CD11b in U937 cells cultured alone, red line representing the expression of CD11b in U937 cells grown in co-culture with MS-5 cells and grey and pink lines representing isotypic controls, respectively.

The effects of stroma on AraC-induced changes in cell cycle progression were next investigated. As shown in Figure 2A, the percentage of sub-G_1_ correlated well with a percentage of annexin-positive cells further confirming that stroma protected from apoptosis induced by high dose AraC. Low-dose AraC (100 nM) decreased proportion of U937 cells in G_1_-phase and caused an accumulation of cells in S-phase (Figure 2A) and these effects were associated with an increase in inhibitory phosphorylation of CDC2/cyclin-dependent kinase 1 (CDK1) (Figure 2B), as previously described (Tomic et al., 2022). The presence of stromal cells prevented the accumulation of U937 in S-phase and decreased the level of Tyr-15-phosphorylated CDC2 after 72 h of incubation. As shown in Figures 2A–C stromal cells-induced decrease in CDC2 phosphorylation paralleled a decrease in the expression of CD11b. Therefore, we confirmed that differentiating effects of low-dose AraC correlate with the cell cycle arrest and CDC2 inhibition and that the presence of stromal cells inhibited differentiation of U937 cells in response to low-dose AraC.

### 3.2 The effects of stromal cells do not depend on mutational status of *p53* and *FLT3* of AML cell lines

**FIGURE 3:**
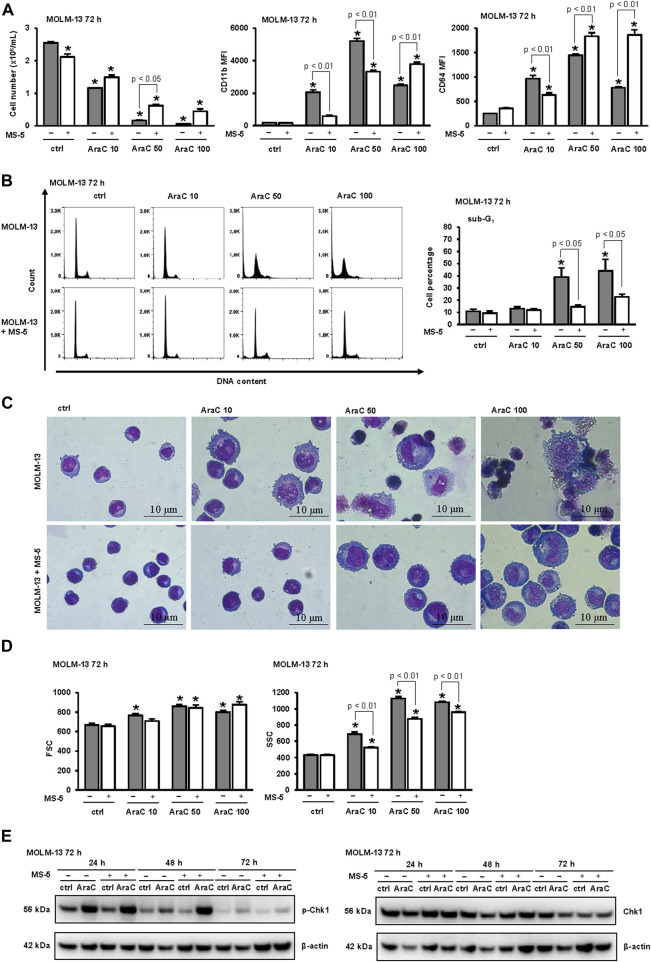
The presence of stromal MS-5 cell line reduces differentiation of MOLM-13 cells induced by low-dose AraC. MOLM-13 cells were cultured either alone or in the presence of MS-5 stromal cell line and untreated (ctrl) or treated with AraC (10, 50, 100 nM). **(A)** The number of viable cells and the expression of differentiation markers 72 h after addition of AraC. Mean fluorescence intensity (MFI) of CD11b and CD64 was calculated as described under “Materials and methods” section. **(B)** Representative histograms from propidium-labeled cells and the percentage of cells in sub-G1. **(C)** Representative May-Grünwald-Giemsa stained cytospin preparations of MOLM-13 cells (×100 magnification). **(D)** Flow cytometry analysis of forward scatter (FSC) and side scatter (SSC) of MOLM-13 cells. **(E)** MOLM-13 cells were cultured either alone or in the presence of MS-5 stromal cell line and untreated (ctrl) or treated with AraC (10 nM). Total cell lysates of cells were isolated after 24, 48 and 72 h and analyzed by Western blotting for the level of Ser-345-phosphorylated Chk1 and total Chk1. Representative immunoblots from three independent experiments are shown. Results are mean ± S.E. (error bars) of at least three independent experiments. *, *p* < 0.05 (ANOVA and Tukey) compared with control (ctrl).

The effects of stroma may depend on the genetic background of the respective leukemia cells. The enhancing effects of stroma on AML differentiation have been reported in AML cells carrying internal tandem duplication-*FLT3* (ITD-*FLT3*) in response to gilteritinib (Sexauer et al., 2012). In addition, cytarabine-mediated differentiation has been reported to depend on the presence of *p53* tumor suppressor (Meyer et al., 2009). Since U937 cell line is *p53*-mutated having wt *FLT3*, we next tested the effects of stroma on MOLM13, which is a wt *p53*-expressing human AML cell line carrying ITD/*FLT3*. As shown in Figure 3, MOLM-13 cells were much more sensitive to the effects of AraC. The number of viable cells was significantly reduced even after incubation with 10 nM AraC (Figure 3A) and the increase in the percentage of sub-G_1_ was observed in samples treated with both 50 and 100 nM AraC (Figure 3B). Cell cycle analysis revealed that AraC-mediated arrest occurred earlier in S-phase, which is probably due to a functional p53 protein (Figure 3B). However, the presence of stromal cells had similar effects by decreasing the expression of CD11b in cells treated with low doses of AraC and preventing cytotoxicity of high doses (Figure 3A). As shown in Figures 3C, D, stromal cells reduced the morphological changes in MOLM-13 cells and inhibited an increase in SSC by flow cytometry analysis. In addition, the presence of stroma reduced the activation of DNA damage signaling pathway as measured by the level of the activating phosphorylation of Chk1 on S345 (Figure 3E). These data confirmed that the inhibitory effects of stroma on differentiation in response to LDAC can be observed in different cell lines irrespective of the presence of *p53* or *FLT3* mutation.

### 3.3 High dose AraC used in the co-culture assays affects the viability and function of the stromal cells themselves

**FIGURE 4:**
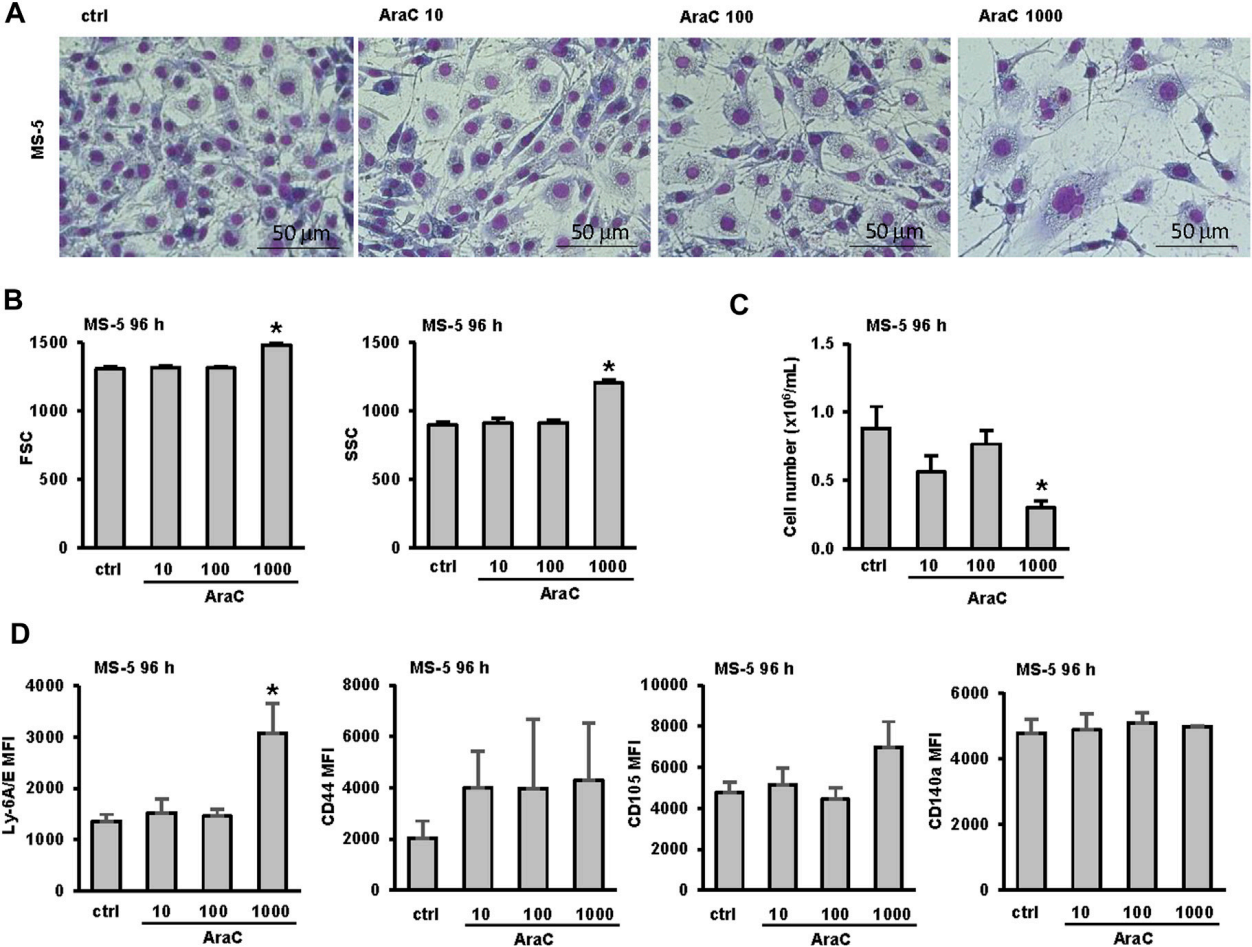
Low-dose AraC used in the co-culture assays does not affect the viability and phenotype of the stromal cells themselves. MS-5 stromal cells were seeded in 6-well plates and AraC (10, 100, 1,000 nM) was added after 24 h. **(A)** Representative May-Grünwald-Giemsa stained MS-5 cells (×40 magnification) 72 h after addition of AraC. **(B)** Flow cytometry analysis of forward scatter (FSC) and side scatter (SSC) of MS-5 cells 72 h after addition of AraC. **(C)** The number of viable MS-5 cells 72 h after addition of AraC. **(D)** Immunophenotype of MS-5 cells. Mean fluorescence intensity (MFI) of Ly-6A/E, CD44, CD105 and CD140a was calculated as described under “Materials and methods” section. Results are mean ± S.E. (error bars) of at least three independent experiments. *, *p* < 0.05 (ANOVA and Tukey) compared with control (ctrl).

Cytarabine is known to induce genotoxicity in primary mesenchymal stromal cells (Gynn et al., 2021). To test whether different doses of AraC used in our study affected MS-5 cells, the stromal cells were stained and analyzed after 72 h of incubation. Morphological analysis demonstrated that the administration of the highest AraC dose (1,000 nM) to MS-5 cells resulted in notable morphological alterations, which were corroborated by flow cytometry analysis and correlated with a substantial decrease in cell viability (Figures 4A–C). However, no significant changes in the number of viable cells, FSC or SSC were detected in MS-5 cells treated with LDAC.

To further test for possible phenotypic changes of MS-5 cells exposed to increasing doses of AraC, flow cytometry analysis of the expression of stromal and hemopoietic markers was performed after 72 h of incubation. MS-5 cells stained positive for conventional mesenchymal stem/stromal cell markers Ly-6A/E (Sca-1) (Morikawa et al., 2009; Houlihan et al., 2012), CD44 (Morikawa et al., 2009; Houlihan et al., 2012; Schelker et al., 2018), CD105 (Morikawa et al., 2009; Houlihan et al., 2012; Schelker et al., 2018), CD140a PDGFR-α, platelet-derived growth factor receptor alpha (Morikawa et al., 2009; Houlihan et al., 2012) (Figure 4D) and negative for CD73, CD34, CD45 and CD11b (data not shown). As shown in Figure 4D, the only significant change in the expression of markers induced by AraC was a significant increase in the expression of Ly-6A/E (Sca-1) upon the treatment with a toxic dose of AraC (1,000 nM).

Based on the obtained data, we concluded that the cytotoxic dosage of AraC impacts both AML and stromal cells. However, the influence of the stromal microenvironment on the differentiation of AML cells induced by LDAC cannot be attributed to the cytotoxic effect of AraC on the stromal cells themselves.

### 3.4 Stroma abolishes AraC-induced activation of cytokine signaling pathways

**FIGURE 5:**
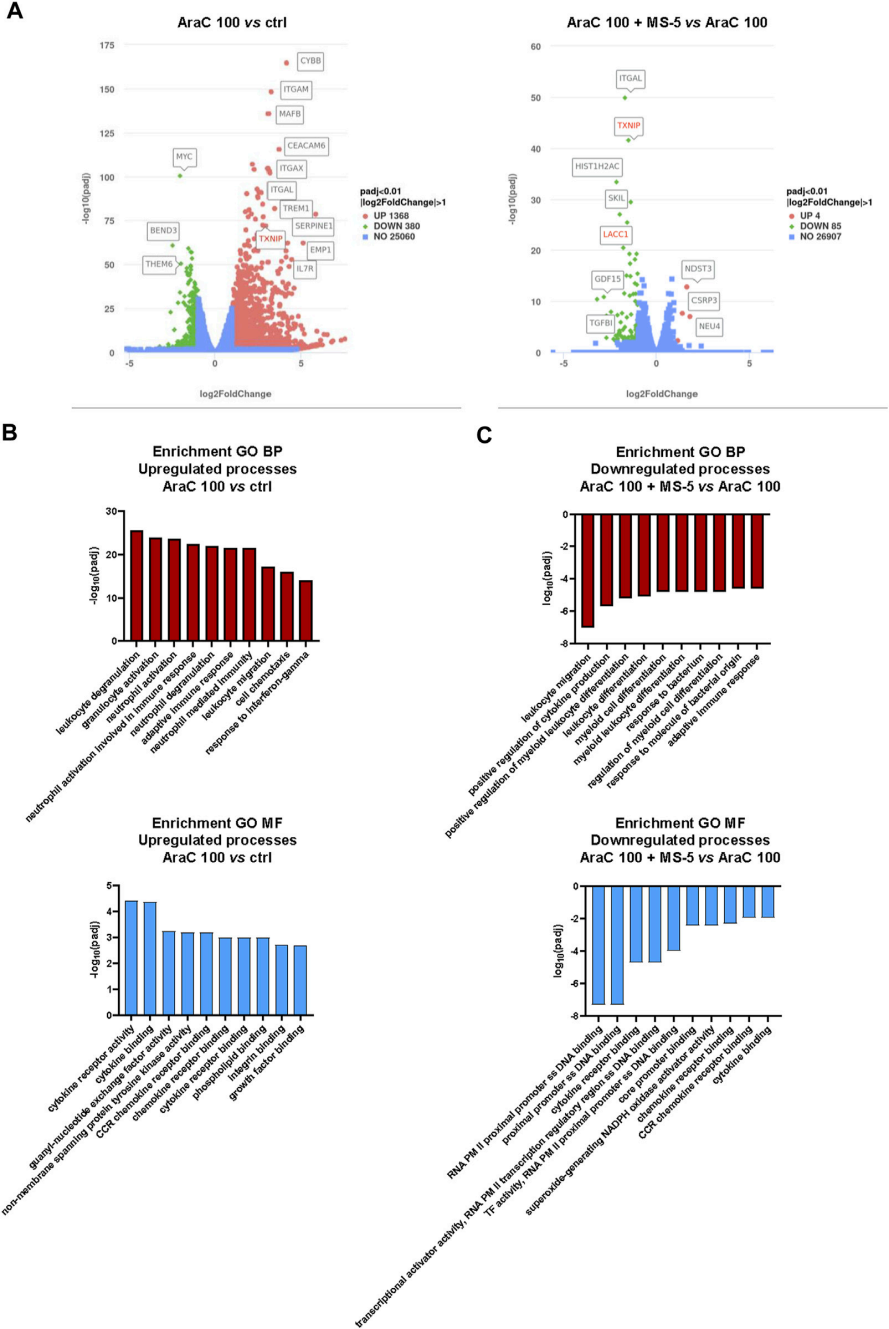
Low-dose AraC induces expression of differentiation-associated genes together with activation of cytokine and chemokine signaling pathways and stromal co-culture inhibits these effects. **(A)** Volcano plots presenting differentially expressed genes in U937 cells treated with AraC (100 nM) for 24 h with or without co-culture with MS-5 stromal cells. **(B,C)** Enriched Gene ontology (GO) pathways associated with biological processes (BP) and molecular functions (MF) in U937 cells treated with AraC (100 nM) for 24 h with or without co-culture with stromal MS-5 cells. Presented values are |log(padj)| in enrichment analysis. Upregulated pathways are presented as positive, while downregulated pathways are presented as negative values.

Transcriptomic analysis of U937 cells exposed to a low-dose of AraC (100 nM) validated our phenotypic observations, revealing significant alterations in gene expression predominantly associated with differentiation markers in cells treated with or without the presence of MS-5 cells (Figure 5A). Notably, genes encoding key differentiation markers, including CD11b (*ITGAM*—coding for CD11b, *CYBB*—coding for p91-phox subunit of NADPH oxidase, *ITGAL*—coding for CD11a, *ITGAX*—coding for CD11c and *MafB*—coding for transcription factor that plays an important role in the regulation of lineage-specific hematopoiesis), exhibited the most pronounced alterations in the expression. Interestingly, *TXNIP*, coding for Thioredoxin Interacting Protein, emerged as one of the highly differentially expressed genes in this experimental model. Previous study has demonstrated its association with the induction of leukemia differentiation (Zeng et al., 2016), and our own research has previously reported an elevation of *TXNIP* expression in U937 cells undergoing differentiation in the presence of a pyrimidine synthesis inhibitor (Dembitz et al., 2020).

To gain a better understanding of the underlying biological processes responsible for AraC-mediated differentiation and inhibitory effects of stroma, we performed enrichment analysis on Gene ontology pathways (Figures 5B and 5C). The signaling pathways showing the most significant upregulation in response to AraC treatment were found to be primarily associated with cytokine signaling and leukocyte activation. However, in the presence of stromal cells, these effects were noticeably attenuated, suggesting a potential link between the suppression of differentiation and the inhibition of cytokine signaling.

### 3.5 Inhibitory effects of MS-5 stromal cells on AML cell differentiation do not depend on cell-to-cell contact and inhibition of CXCL12 and TGF-β

**FIGURE 6:**
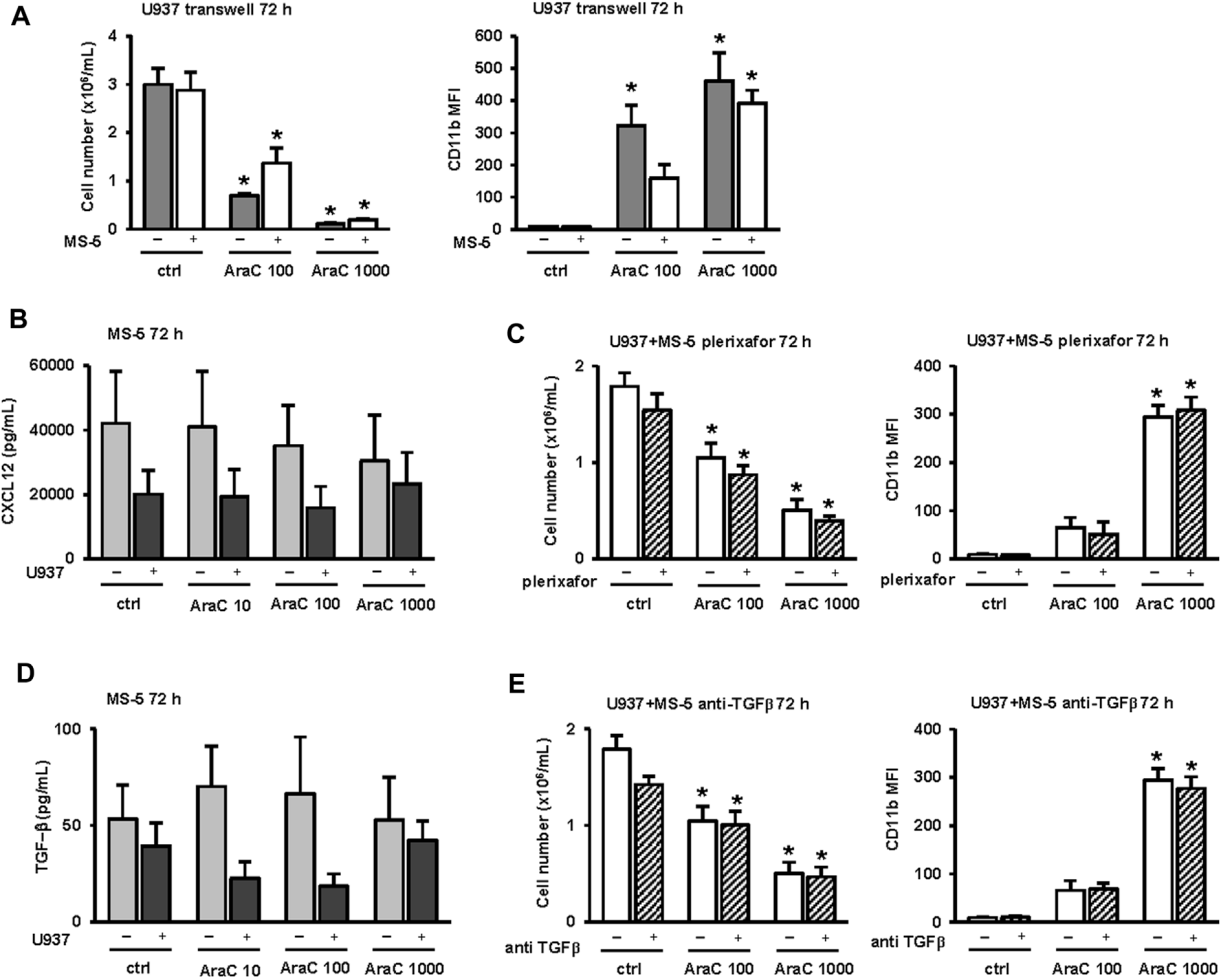
Inhibitory effects of MS-5 stromal cells on AML cell differentiation do not depend on cell-to-cell contact and inhibition of CXCL12 and TGF-β. **(A)** The number of viable cells and the expression of CD11b in U937 cells that were plated in a 0.4 μm porous transwell inserts in the presence or absence of MS-5 monolayers and untreated (ctrl) or treated with AraC (100, 1,000 nM) for 72 h **(B)** LEGENDplexTM multi-analyte flow assay of CXCL12 in supernatants of MS-5 cells that were cultured alone or in the presence of U937 cells and untreated (ctrl) or treated with AraC (10, 100, 1,000 nM) for 24 h. **(C)** The effects of plerixafor (20 μM) on the number of viable U937 cells and the expression of CD11b in U937 cells that were cultured in the presence of MS-5 monolayers and untreated (ctrl) or treated with AraC (100, 1,000 nM) for 72 h **(D)** LEGENDplexTM multi-analyte flow assay of TGF-β in supernatants of MS-5 cells that were cultured alone or in the presence of U937 cells and untreated (ctrl) or treated with AraC (10, 100, 1,000 nM) for 24 h. **(E)** The effects of anti-TGF-β (0.5 μg/mL) on the number of viable cells and the expression of CD11b in U937 cells that were cultured in the presence of MS-5 monolayers and untreated (ctrl) or treated with AraC (100, 1,000 nM) for 72 h. Results are mean ± S.E. (error bars) of at least three independent experiments. *, *p* < 0.05 (ANOVA and Tukey) compared with control (ctrl).

To test first whether the effects of MS-5 cells on AML cell differentiation depend on cell-to-cell contact, U937 cells were plated in a 0.4 μm porous transwell inserts in the presence or absence of MS-5 monolayers. As shown in Figure 6A, in the presence of transwell inserts, no protective effects of stroma on the number of viable cells were observed upon treatment with a cytotoxic dose of AraC (1,000 nM). However, the use of transwell did not prevent the effects of MS-5 cells on U937 cells treated with 100 nM AraC, suggesting that inhibitory effects on differentiation in response to LDAC do not require direct contact with stromal cells.

To test for the possible role of soluble factors, the levels of principal mouse HSC myeloid cytokines were measured in supernatants of MS-5 cells incubated with or without U937 cells for 72 h. As shown in Figure 6B, the high levels of SDF-1, also known as chemokine CXCL12, were detected in supernatants. CXCL12 is known to bind to its receptor, C-X-C chemokine receptor type 4 (CXCR4), and U937 cells have been shown to express high levels of CXCR4 (Tavor et al., 2008; Zeng et al., 2009). To analyze the effects of CXCR4 inhibition on AML cells growth and differentiation, we used the antagonist plerixafor (AMD3100) at a dose that was previously shown to induce the expression of CD15 on U937 cells after 9 days of incubation (Tavor et al., 2008). As shown in Figure 6C, the addition of plerixafor had no significant effects on the number of viable U937 cells or the expression of CD11b when cells were treated with two doses (100 and 1,000 nM) of AraC in co-culture with MS-5.

To test for the possible role of TGF-β1 in stroma-mediated effects (Figure 6D), the anti-mouse TGF-β1 antibodies were added in co-culture of U937 and MS-5 cells. As shown in Figure 6E, the addition of blocking antibodies had no significant effects on the number of viable U937 cells and the level of CD11b.

Therefore, we concluded that the inhibitory effects of stroma on U937 differentiation induced by LDAC do not depend on either TGF-β1 or the functional CXCL12/CXCR4 axis.

### 3.6 The presence of MS-5 stromal cells does not reduce the level of reactive oxygen species (ROS) in U937 cells treated with LDAC

**FIGURE 7:**
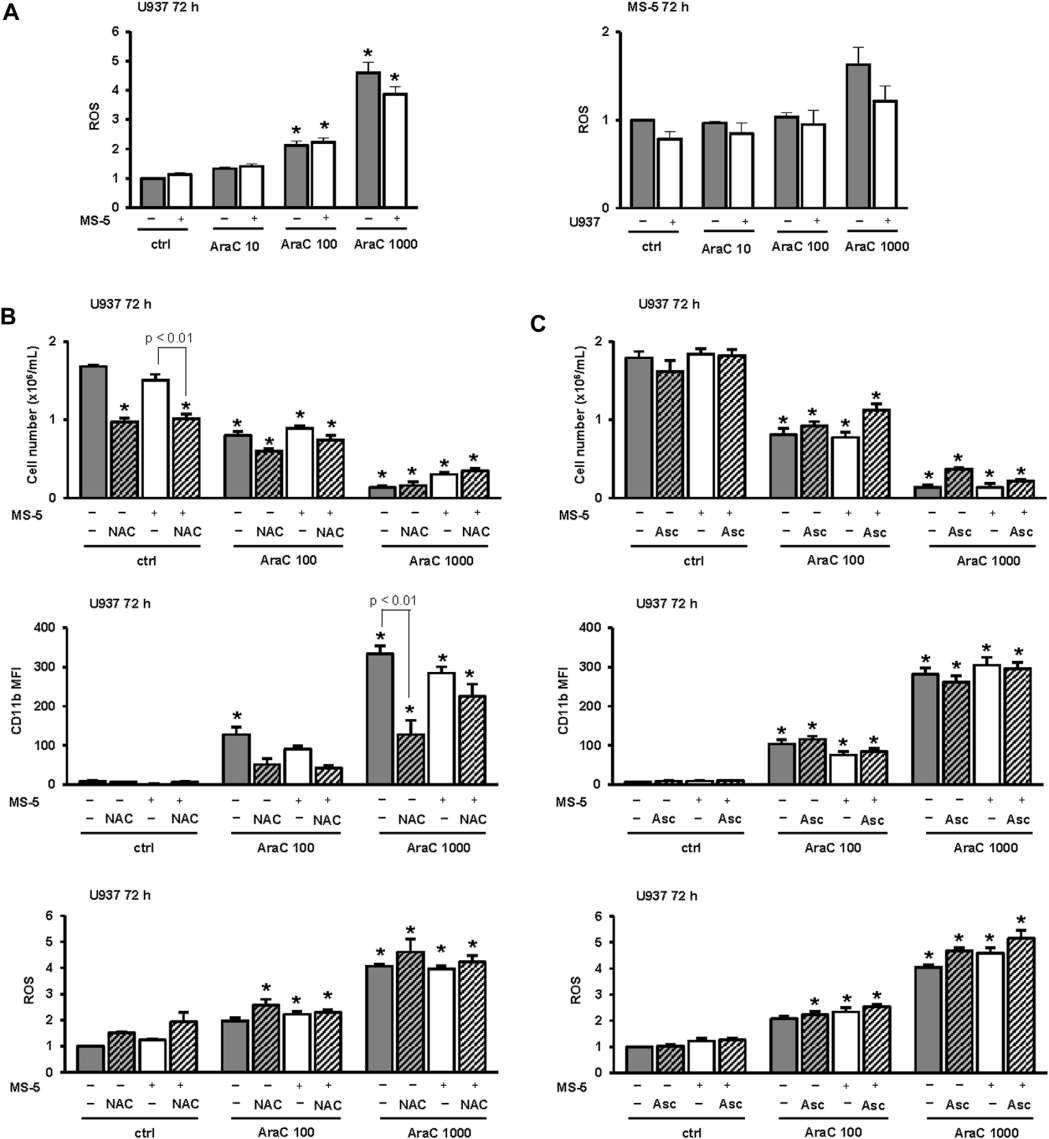
Inhibitory effects of MS-5 stromal cells on AML cell differentiation do not depend on the level of reactive oxygen species (ROS). **(A)** The level of ROS in U937 cells and MS-5 cells that were plated alone or in the co-culture and untreated (ctrl) or treated with AraC (10, 100, 1,000 nM) for 72 h. **(B)** The number of viable U937 cells, the expression of CD11b and the level of ROS in U937 cells that were cultured either alone or in the presence of MS-5 stromal cell line and untreated (ctrl) or treated with AraC (100, 1,000 nM) for 72 h. N-Acetyl-L-cysteine (NAC) (10 mM) was added 15 min before the addition of AraC. **(C)** The number of viable U937 cells, the expression of CD11b and the level of ROS in U937 cells that were cultured either alone or in the presence of MS-5 stromal cell line and untreated (ctrl) or treated with AraC (100, 1,000 nM) for 72 h. Ascorbic acid (Asc) (500 μM) was added 15 min before the addition of AraC and again after 48 h *, *p* < 0.05 (ANOVA and Tukey) compared with control (ctrl).

Our transcriptomic data revealed an increase in the expression of *TXNIP* in U937 cells treated with 100 nM AraC, and the expression was reduced by the presence of stromal cells. As the increased expression of TXNIP was associated with an increased level of ROS as a driver of differentiation (Zeng et al., 2016), we measured the level of ROS in U937 cells and MS-5 cells incubated either alone or in the co-culture. As shown in Figure 7A, the level of ROS dose-dependently increased in U937 cells treated with AraC. In MS-5 cells, an increase in the level of ROS was measured only in the presence of the highest dose. However, there was no statistically significant difference in the level of ROS measured in LDAC-treated U937 cells when cultured alone or in the presence of stromal cells suggesting that the effect of stroma on U937 cell differentiation is not mediated by regulation of ROS levels.

To further test for the role of ROS, N-acetyl cysteine (NAC) was added to the culture at the same concentration that was previously showed to attenuate ROS generation induced by short-term treatment with oxidant H_2_O_2_ (Wang et al., 2020). As shown in Figure 7B, NAC reduced the number of viable U937 control cells treated alone or in the co-culture with MS-5 cells. When we tested for the effects on the expression of CD11b, the addition of NAC reduced the expression of CD11b in both U937 cells treated alone or in the co-culture. However, the addition of NAC had no inhibitory effects on the level of ROS in any of the groups tested as measured by DHR123 uptake, suggesting that AraC generates ROS that are superoxide species and that vitamin C (ascorbic acid) may be more effective in attenuating ROS generation by AraC (Schneider et al., 2005). Therefore, we tested the effects of ascorbic acid applied at 100 μM and 500 μM that is within the range of concentrations that were previously shown to exert the effects in AML cell lines (Liu et al., 2020). However, as shown in Figure 7C, even the addition of 500 μM ascorbic acid did not reduce ROS in U937 cells after 72 h. Additionally, and unlike NAC, ascorbic acid did not reduce the expression of CD11b (Figure 7C) and the same effects were observed after addition of 100 μM ascorbic acid (data not shown).

Therefore, we concluded that the inhibitory effects of stroma on the expression of differentiation markers in response to LDAC cannot be ascribed to a decrease in the level of ROS.

### 3.7 The presence of stromal cells reduces expression of differentiation markers induced by low-dose AraC in primary samples from AML-M4 and MDS/AML patients

**FIGURE 8:**
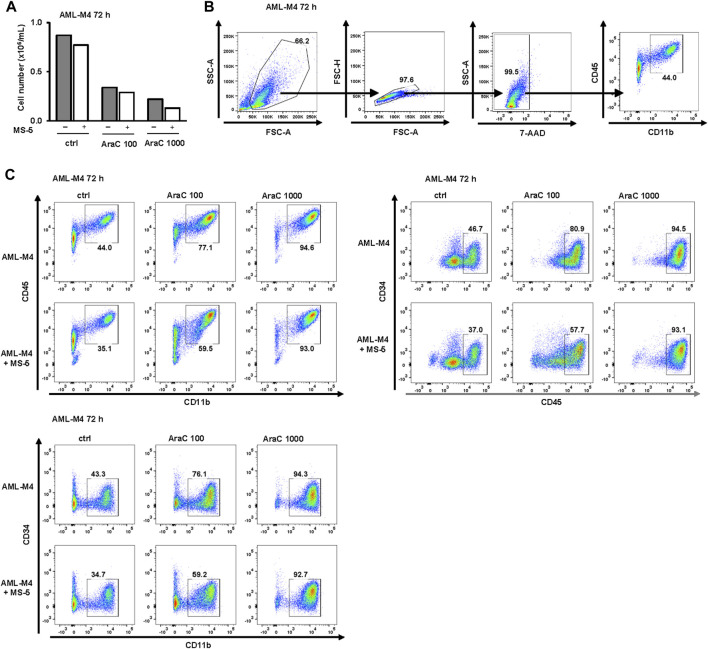
Inhibitory effects of MS-5 stromal cells on AraC-induced expression of differentiation markers in a primary sample from AML-M4. Non-adherent mononuclear cells from bone marrow sample (Pt 07, normal karyotype, *FLT3*-ITD, *NPM*/mut) were plated at concentration 0.4 × 10^6^/mL in medium supplemented with 50 ng/mL IL-3, IL-6, SCF and FLT3L and incubated with AraC (100, 1,000 nM) in the presence or absence of MS-5 cells. **(A)** The number of viable cells was determined by trypan blue exclusion. **(B)** Gating strategy. **(C)** Flow cytometric analysis of CD11b^+^CD45^+^, CD45^+^CD34^−^ and CD11b^+^CD34^−^ populations. Percentage of cells in the population of interest is indicated in respective gates.

As shown in Figure 8, in a primary sample from AML patient with acute myelomonocytic leukemia (AML-M4), the viability of cells decreased and the expression of the differentiation marker CD11b increased in a dose-dependent manner upon treatment with AraC. Moreover, the population of CD45^high^CD34^-^ cells, as well as the FSC and SSC parameters, exhibited an increase, providing further evidence of blast maturation. Similar to the effects observed in U937 and MOLM-13 cell lines, the presence of MS-5 cells led to a reduction in differentiative properties.

**FIGURE 9:**
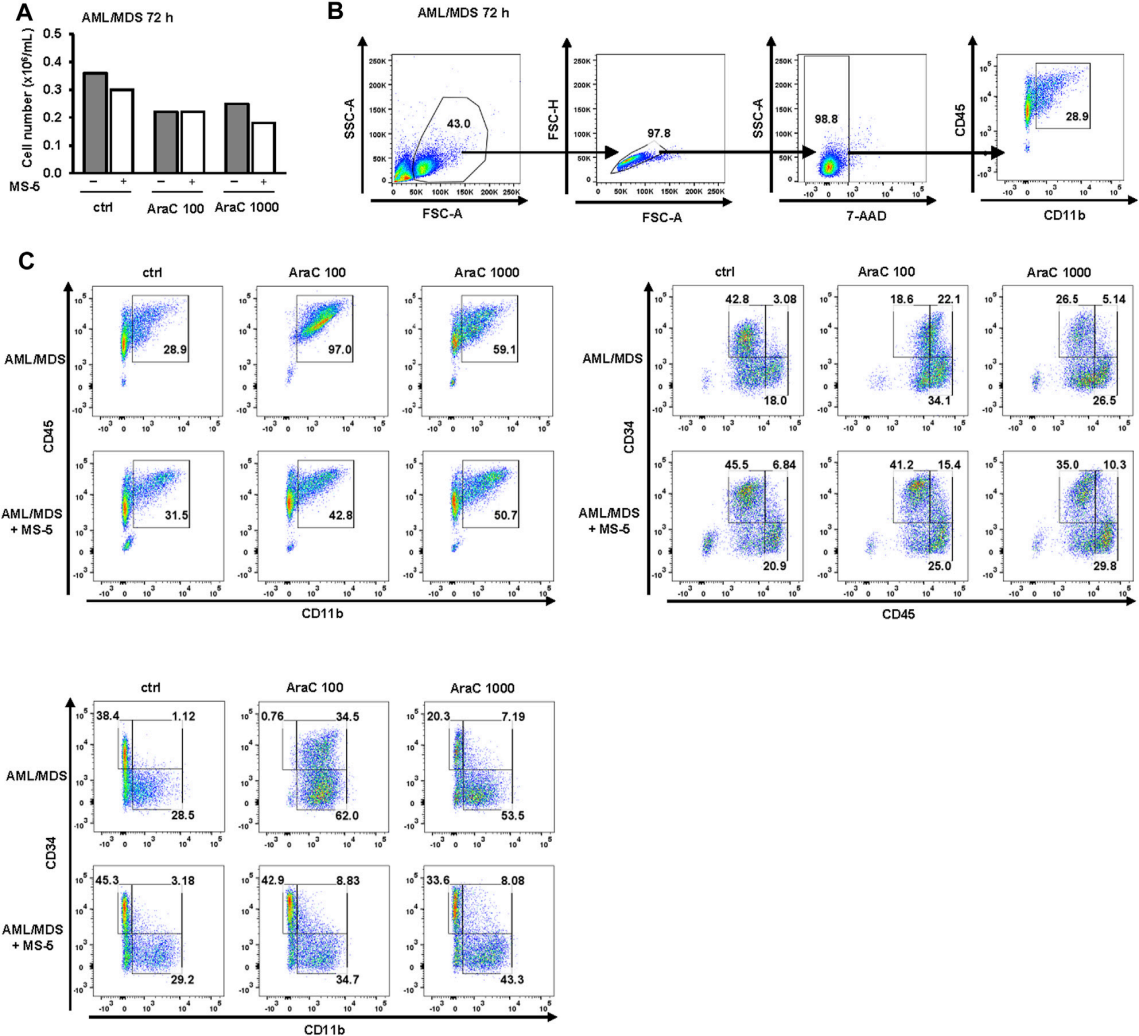
Inhibitory effects of MS-5 stromal cells on AraC-induced expression of differentiation markers in a primary sample from AML/MDS. Non-adherent mononuclear cells from bone marrow sample (Pt 09, secondary AML progressed from MDS, normal karyotype) were plated at a concentration of 0.4 × 10^6^/mL in medium supplemented with 50 ng/mL IL-3, IL-6, SCF and FLT3L and incubated with AraC (100, 1,000 nM) in the presence or absence of MS-5 cells. **(A)** The number of viable cells was determined by trypan blue exclusion. **(B)** Gating strategy. **(C)** Flow cytometry analysis for CD11b^+^CD45^+^; CD45^low^CD34^+^, CD45^high^CD34^+^, CD45^high^CD34^-^; CD11b^−^CD34^+^, CD11b^+^CD34^+^, CD11b^+^CD34^−^ populations. Percentage of cells in the population of interest is indicated in respective gates.

In a primary sample obtained from an AML patient with a history of progression from MDS (AML/MDS, Figure 9), similar to AML-M4, treatment with AraC induced an upregulation of CD11b. Furthermore, a shift from the CD34^+^CD45^low^ and CD11b^−^CD34^+^ immature blast phenotype in control cells towards the more mature CD45^high^CD34^+^ and CD11b^+^CD34^+^ phenotypes, and subsequently to the CD45^high^CD34^-^ and CD11b^+^CD34^−^ phenotypes, was observed. Notably, co-culturing with MS-5 cells resulted in an increase in immature CD34^+^ cells and a decrease in mature CD11b^+^ and CD45^high^ cells, as well as a reduction in SSC, indicating stromal cell-mediated inhibition of differentiation.

## 4 Discussion

The mode of action of LDAC in the treatment of the myelodysplastic syndromes and AML remains unclear. Despite the demonstrated capacity of AraC to induce differentiation of AML cells *in vitro*, the phenomenon of differentiation is seldom observed *in vivo*. In elderly patients, the induction of remission by LDAC is often accompanied by a hypoplastic or aplastic phase, implying a direct cytotoxic mechanism of action. However, the absence of aplasia and the gradual appearance of more mature myeloid cells harboring original karyotypic abnormalities are arguments in favor of a differentiating effect (Reilly et al., 1987; Degos, 1990). In this study, we used an *in vitro* model that mimics the conditions of bone marrow environment, allowing for direct interactions between leukemia and MS-5 stromal cells, which were previously shown to be the most supportive for AML (Griessinger et al., 2014). Previous studies have established that MS-5 cells possess the ability to mitigate the cytotoxic effects of AML cell lines, such as U937 cells, when exposed to high doses of AraC (500–3,000 nM) (Konopleva et al., 2002; Moschoi et al., 2016; Di Tullio et al., 2017). However, the impact of lower doses of AraC on the induction of differentiation has not yet been explored in this context. The present study is the first to report that the differentiation of AML cell lines and primary blasts induced by low-dose AraC is inhibited by the presence of bone marrow stromal cells. It is noteworthy that the diminished differentiation observed in the stromal co-culture system, as compared to the effects observed in suspension cultures with LDAC, likely aligns more closely with the clinical responses observed in patients.

Our recent study revealed that LDAC-induced differentiation of AML cell lines and primary blasts in suspension cultures depends on cell cycle arrest and activation of DNA damage signaling pathway (Tomic et al., 2022). These findings are consistent with the results obtained in the present study, which demonstrate that stromal cells effectively diminish LDAC-induced S-phase arrest, CDC2 inhibition, CHK1 activation, concurrent with a decrease in CD11b expression. Notably, the inhibitory effects of stromal cells were observed in both U937 and MOLM-13 cell lines, indicating that their impact is independent of *p53* and *FLT3* mutational status in AML. Moreover, this similar stromal effect was also observed in two distinct primary samples, one representing AML-M4 with *FLT3*-ITD and the other representing AML/MDS, both of which proliferated in liquid suspension cultures and displayed responsiveness to LDAC. Hence, while the precise factors governing the sensitivity to cytarabine-induced differentiation remain unidentified, our findings suggest that stromal cells possess the ability to attenuate differentiation irrespective of AML subtypes and genetic abnormalities.

The exact mechanism responsible for stroma-mediated inhibition of AML differentiation in response to LDAC remains unclear. For AraC-mediated cytotoxicity, several mechanisms have been proposed to be responsible for the protective effect of stroma, and majority of studies focused on the role of adhesion molecules and cytokines (Kokkaliaris and Scadden, 2020). The antiapoptotic effects of MS-5 cells on NB4 and HL-60 cell lines exposed to 1,000 nM AraC were reproduced by conditioned medium derived from MS-5 cells, but the specific soluble factor remained unidentified, and the transwell experiments did not completely exclude possible dependence on direct contact, particularly in certain primary AML samples (Konopleva et al., 2002). Among the multitude of cytokines generated by stromal cells, the cytokine CXCL12 stands out as the cytokine identified to exert an inhibitory effect on the differentiation of U937 cells (Tavor et al., 2008). Stromal cells, including MS-5, secrete SDF-1 or CXCL12 (Li et al., 2014), U937 cells express high levels of a ligand, CXCR4 (Tavor et al., 2008; Zeng et al., 2009), and disruption of CXCL12/CXCR4 by plerixafor (AMD3100) renders AML cells more sensitive to cytotoxic effects of AraC in mice (Nervi et al., 2009). In the current study, CXCL12 was detected in the supernatants of MS-5 cells. However, the addition of plerixafor, a CXCL12 receptor antagonist, did not impede the inhibitory effects of stroma on CD11b expression. Moreover, plerixafor had no substantial impact on the viability of cells treated with 1,000 nM AraC in co-cultures over a period of 3 days. In a study conducted by Tavor et al. (2008) it was observed that treatment of U937 cells with 10 μM AMD3100 resulted in an upregulation of CD15 expression after 9 days of *in vitro* treatment. However, it is important to note that we did not assess CD15 expression following extended incubation periods. Notably, during the time when we evaluated the inhibitory effects of stroma in our co-culture experiments, no increase in CD11b expression was observed in control cells. Although we cannot rule out the possibility that the concentration of plerixafor was not sufficient to completely prevent the effect of SDF-1, our findings align with a similar model involving primary blasts co-cultured with human mesenchymal cells, where plerixafor demonstrated no significant effects on cytarabine sensitivity. Notably, in this model, the introduction of antibodies against TGF-β1 resulted in increased proliferation of primary AML cells; however, this increase did not reach statistical significance in terms of the number of cells treated with cytarabine (Schelker et al., 2018). Importantly, our study reveals that the addition of anti-TGF-β1 antibodies did not elicit any significant effects on the differentiation and cytotoxicity of U937 cells when grown in co-culture with MS-5 cells. However, it is possible that the effects of cytokines secreted by murine stromal cells might differ from the one we would see if human stromal support, i.e., HS-5 cell line, was used.

More recent studies have focused more on the metabolic aspect of stromal support in AML. Several studies have demonstrated that AML cells rely on oxidative phosphorylation as a mechanism of chemotherapy resistance, and their bioenergetic capacity is enhanced in the presence of stromal cells (Forte et al., 2020). Notably, U937 cells adherent to MS-5 cells exhibited an enhanced resistance to 3 μM Ara-C, which was correlated with the uptake of mitochondrial markers from the stroma (Moschoi et al., 2016). This uptake was accompanied by an increase in mitochondrial transfer and oxidative phosphorylation, resulting in elevated levels of ROS. Importantly, the level of ROS has been shown to play an important role in determining the survival of AML cells following chemotherapy (van Gastel et al., 2020; Cai et al., 2022). In our model, the level of ROS increased in Ara-C treated cells, as previously described (Hosseini et al., 2019; Forte et al., 2020). However, we observed that the presence of MS-5 cells led to a slight reduction in ROS levels specifically in cells exposed to cytotoxic doses of AraC. Notably, this reduction was not observed in cells undergoing differentiation induced by AraC without exhibiting cytotoxic effects. In the presence of stroma, the inhibitory effects of NAC on AraC-induced differentiation were not observed. Surprisingly, despite the addition of NAC, the level of ROS did not decrease. The similar lack of the effects of NAC on ROS levels have been previously described in U937 cells in which the same concentration of NAC was completely capable of attenuating ROS generation induced by short-term treatment with oxidant H_2_O_2_ (Wang et al., 2020). Even the addition of ascorbic acid, which was previously shown to reduce the level of ROS in two of the four lines tested after 48 h (Liu et al., 2020), did not affect the level of ROS in U937 cells after 72 h. While the inhibitory effect of NAC on CD11b expression has been documented in other cell types (Roy et al., 2008), our observations of its lack of the effects on reactive ROS levels, along with its antiproliferative effects when administered alone, indicate that the inhibitory effects of NAC on CD11b expression may be mediated by mechanisms distinct from ROS regulation.

In the current study, we did not assess changes in the level of metabolites in the culture media following co-culture, nor did we investigate the potential regulation of drug metabolizing enzymes in the stromal cells. BM stromal cells are known to provide aspartate for pyrimidine synthesis allowing cells to repair DNA damage induced by AraC, a pyrimidine analog (van Gastel et al., 2020), and our previous research has identified a shared mechanism between cytarabine-induced differentiation and pyrimidine synthesis inhibitors (Dembitz et al., 2019; Tomic et al., 2022). The presence of BM stromal cells has been shown to reduce ATRA-mediated differentiation through the expression of retinoid-inactivating enzymes (Su et al., 2015), and it is known that AraC treatment increases the expression of drug metabolizing enzymes within the leukemic BM microenvironment (Su et al., 2019). The maintenance and proliferation of hematopoietic stem cells is known to be supported by hypoxia inducible factor (HIF) within the bone marrow (Takubo et al., 2010), and the induction of HIF1α is known to reduce the cellular uptake of AraC in AML cell lines (Jin et al., 2009). Therefore, the effects of stromal cells on differentiation may also result from the metabolism and sequestration of AraC, leading to a reduction in its effective concentration under co-culture conditions.

In summary, the results of our study show that the presence of stromal cells reduces the expression of differentiation markers induced by LDAC and that the few cells that survive cytotoxic doses continue to express CD11b. Although there are many studies investigating the mechanisms by which stromal cells protect AML cells from cytotoxicity, there are very few studies that determine the expression of differentiation markers in living cells during or after AraC treatment (Moschoi et al., 2016). These surviving cells, often referred as resistant (Moschoi et al., 2016; Di Tullio et al., 2017; Hosseini et al., 2019), residual (van Gastel et al., 2020), or senescent (Duy et al., 2021), are believed to contribute to disease relapse. It is possible that many of these cells exhibit partial differentiation but retain the ability to undergo de-differentiation, similar to what has been observed in cells differentiated by ATRA (McKenzie et al., 2019). Further research is needed to better understand the mechanisms of differentiation and de-differentiation in these cells and their role in disease progression and relapse.

## Data Availability

The datasets presented in this study can be found in online repositories. The names of the repository/repositories and accession number(s) can be found below: https://www.ebi.ac.uk/arrayexpress/, E-MTAB-13192.
