# Supplementary material for: Bone marrow stromal cells reduce low-dose cytarabine-induced differentiation of acute myeloid leukemia
Source: Front Pharmacol. 2023 Oct 26;14:1258151. doi: 10.3389/fphar.2023.1258151 (PMC10637411; doi:10.3389/fphar.2023.1258151)
Supplement: Supplementary file 1 [file DataSheet1.docx]

Supplementary Material

Bone marrow stromal cells reduce low-dose cytarabine-induced differentiation of acute myeloid leukemia

Tomislav Smoljo, Barbara Tomic, Hrvoje Lalic, Vilma Dembitz, Josip Batinic, Antonio Bedalov, Dora Visnjic*

* Correspondence:

Dora Visnjic

visnjic@mef.hr

# Supplementary Table 1. Reagents and resources used

| **Reagent or RESOURCE** | **Source** | **Identifier** |
| --- | --- | --- |
| **Antibodies** | | |
| IgG1-FITC (679.1Mc7) | Immunotech BeckmanCoulter, Marseille, France | A07795, RRID:AB_2832964 |
| IgG1-FITC (MOPC-21) | BioLegend, San Diego, CA, USA | 400110, RRID:AB_2861401 |
| IgG2a-FITC (RTK2758) | BioLegend, San Diego, CA, USA | 400505, RRID:AB_2736919 |
| IgG2b-FITC (RTK4530) | BioLegend, San Diego, CA, USA | 400606, RRID:AB_326550 |
| IgG1-APC (MOPC-21) | BD Biosciences, San Jose, CA, USA | 555751, RRID:AB_398613 |
| IgG2b-PE (RTK4530) | BioLegend, San Diego, CA, USA | 400608, RRID:AB_326552 |
| IgG1-PE (RTK2071) | BioLegend, San Diego, CA, USA | 400407, RRID:AB_326513 |
| IgG1-PE (MOPC-21) | BioLegend, San Diego, CA, USA | 400114, RRID:AB_326435 |
| IgG1-PerCP (559425) | BD Biosciences, San Jose, CA, USA | 559425, RRID:AB_397240 |
| IgG2a-PE/Cyanine7 (RTK2758) | BioLegend, San Diego, CA, USA | 400522, RRID:AB_326542 |
| IgG2a-APC (RTK2758) | BioLegend, San Diego, CA, USA | 400511, RRID:AB_2814702 |
| IgG2a-eFluor 660 (eBR2a) | Thermo Fisher Scientific, Waltham, MA, USA | 50-4321-82, RRID:AB_10598503 |
| CD11b-FITC (Bear1) | Immunotech BeckmanCoulter, Marseille, France | IM0530, RRID:AB_130987 |
| CD11b-FITC (ICRF44) | BioLegend, San Diego, CA, USA | 301330, RRID:AB_2561703 |
| CD11b-PE (M1/70) | BioLegend, San Diego, CA, USA | 101208, RRID:AB_312791 |
| CD64-FITC (22) | Immunotech BeckmanCoulter, Marseille, France | B49185 |
| CD34-APC (8G12) | BD Biosciences, San Jose, CA, USA | 345804, RRID:AB_2686894 |
| CD45-PerCP (2D1) | BD Biosciences, San Jose, CA, USA | 345809, RRID:AB_2868830 |
| CD45-FITC (30-F11) | BioLegend, San Diego, CA, USA | 103108, RRID:AB_312973 |
| CD45-PE (2D1) | BioLegend, San Diego, CA, USA | 368510, RRID:AB_2566370 |
| Ly-6A/E (Sca-1)-FITC (W18174A) | BioLegend, San Diego, CA, USA | 160907, RRID:AB_2910335 |
| CD44-PE (IM7) | BioLegend, San Diego, CA, USA | 103024, RRID:AB_493687 |
| CD73-PE (TY/11.8) | BioLegend, San Diego, CA, USA | 127205, RRID:AB_1089065 |
| CD105-PE/Cyanine7 (MJ7/18) | BioLegend, San Diego, CA, USA | 120409, RRID:AB_1027702 |
| CD140a-APC (APA5) | BioLegend, San Diego, CA, USA | 135907, RRID:AB_2043969 |
| CD34-eFluor 660 (RAM34) | Thermo Fisher Scientific, Waltham, MA, USA | 50-0341-82, RRID:AB_10596826 |
| Chk1 Mouse mAb (2G1D5) | Cell Signaling Technology, Beverly, MA, USA | 2360, RRID:AB_2080320 |
| p-Chk1 (S345) Rabbit mAb (133D3) | Cell Signaling Technology, Beverly, MA, USA | 2348,  RRID:AB_331212 |
| cdc2 Mouse mAb  (POH1) | Cell Signaling Technology, Beverly, MA, USA | 9116,  RRID:AB_2074795 |
| Phospho-cdc2 (Tyr15) antibody | Cell Signaling Technology, Beverly, MA, USA | 9111, RRID:AB_331460 |
| Monoclonal anti-β-actin antibody produced in mouse (AC-15) | Sigma, St. Louis, MO, USA | A5441, RRID:AB_476744 |
| Anti-mouse IgG, HRP-linked antibody | Cell Signaling Technology, Beverly, MA, USA | 7076, RRID:AB_330924 |
| Anti-rabbit IgG, HRP-linked antibody | Cell Signaling Technology, Beverly, MA, USA | 7074, RRID:AB_2099233 |
| TGF-beta 1 Antibody | R&D Systems, Inc., Minneapolis, MN, USA | MAB2402, RRID:AB_358119 |
| Human TruStain FcX™ Fc Receptor Blocking solution | BioLegend, San Diego, CA, USA | 422302, RRID:AB_2818986 |
| TruStain FcX™ PLUS (anti-mouse CD16/32) Antibody | BioLegend, San Diego, CA, USA | 156604, RRID:AB_2783138 |
| **Cell culture** | | |
| RPMI Medium 1640 | Life Technologies, Grand Island, NY, USA | 42401-018 |
| Alpha MEM Eagle | PAN-Biotech, Aidenbach, Germany | P04-21150 |
| Fetal bovine serum (FBS) | Life Technologies, Grand Island, NY, USA | 10270-106 |
| L-Glutamine 200mM | Life Technologies, Grand Island, NY, USA | 25030-024 |
| Sodium pyruvate | Sigma, St. Louis, MO, USA | S8636 |
| Penicillin/streptomycin | Life Technologies, Grand Island, NY, USA | 15070-063 |
| Transwell inserts | Corning®, New York, NY, USA | CLS3450-24EA |
| **Cell lines** | | |
| U937 | ECACC, Salisbury, UK | 85011440 |
| MOLM-13 | a gift from P. Gallipoli (obtained from Sanger Institute) | Cell Models Passport |
| MS-5 | DSMZ, Braunschweig, Germany | ACC 411, RRID:CVCL_2128 |
| **Chemicals, peptides and recombinant proteins** | | |
| 1-β-D-Arabinofuranosylcytosine | Sigma, St. Louis, MO, USA | 251010 |
| N-Acetyl-L-cysteine | Sigma, St. Louis, MO, USA | A7250 |
| Ascorbic acid | Sigma, St. Louis, MO, USA | A5960 |
| Plerixafor | MedChemExpress, Monmouth Junction, NJ, USA | HY-10046 |
| Dimethyl sulfoxide (DMSO) | Honeywell, Muskegon, MI, USA | 472301 |
| Bovine serum albumin (BSA) | Sigma, St. Louis, MO, USA | A4503 |
| Tween® 20 | Sigma, St. Louis, MO, USA | P7949 |
| Sodium azide | Sigma, St. Louis, MO, USA | S8032 |
| Sodium chloride | Fagron, Krakow, Poland | 1002476 |
| Glycine | Sigma, St. Louis, MO, USA | G7126 |
| Sodium hydrogen phosphate dihydrate | Kemika, Zagreb, Croatia | 1410207 |
| Sodium dihydrogen phosphate dihydrate | Kemika, Zagreb, Croatia | 1406707 |
| Dihydrorhodamine 123 (DHR123) | Thermo Fisher Scientific, Waltham, MA, USA | D632 |
| Propidium iodide | Sigma, St. Louis, MO, USA | P4170 |
| Igepal CA | Sigma, St. Louis, MO, USA | I3021 |
| Annexin Binding Buffer 10X | bioWORLD, Dublin, OH, USA | 21720002-1 |
| Cell lysis buffer (10x) | Cell Signaling Technology, Beverly, MA, USA | 9803S |
| Phenylmethylsulfonyl fluoride (PMSF) | Sigma, St. Louis, MO, USA | P7626 |
| Microcystin-LR | Enzo, Life Sciences, Farmingdale, NY, USA | ALX-350-012-C100 |
| Bradford reagent | Sigma, St. Louis, MO, USA | B6916 |
| Bromphenol blue | Sigma, St. Louis, MO, USA | B6131 |
| Trizma® base | Sigma, St. Louis, MO, USA | T1503 |
| Triton X-100 | Sigma, St. Louis, MO, USA | X-100 |
| RNAse | Sigma, St. Louis, MO, USA | R6513 |
| NuPAGE™ LDS Sample Buffer (4X) | Thermo Fisher Scientific, Waltham, MA, USA | NP0007 |
| NuPAGE™ 4 to 12%, Bis-Tris, 1.0–1.5 mm, Mini Protein Gels | Thermo Fisher Scientific, Waltham, MA, USA | NP0323BOX |
| PageRuler™ Plus Prestained Protein Ladder, 10 to 250 kDa | Thermo Fisher Scientific, Waltham, MA, USA | 26619 |
| Amersham™ Protran® Western blotting membrane, nitrocellulose | GE Healthcare Life Sciences, Chicago, IL, USA | GE10600007 |
| Immobilon®-P PVDF Membrane | Millipore | IPVH00005 |
| SuperSignal™ West Pico PLUS Chemiluminescent Substrate | Thermo Fisher Scientific, Waltham, MA, USA | 34577 |
| NycoPrep 1.077 solution | Axis-Shield PoC AS , Oslo, Norway | 1114741 |
| 7-AAD staining solution | Miltenyi Biotec GmbH, Bergisch Gladbach, Germany | 130-111-568 |
| Trypan blue stain (0.4%) | Gibco, Life Technologies, Grand Island, NY, USA | 15250-061 |
| Giemsa solution | Kemika, Zagreb, Croatia | 0710304 |
| May-Grünwald's solution | Kemika, Zagreb, Croatia | 1322804 |
| Annexin A5-FITC | BioLegend, San Diego, CA, USA | 640945 |
| Interleukin-6 human | Sigma, St. Louis, MO, USA | H7416 |
| Interleukin-3 human | Sigma, St. Louis, MO, USA | H7166 |
| FLT3 ligand human | Sigma, St. Louis, MO, USA | H5416 |
| Stem cell factor human | Sigma, St. Louis, MO, USA | H8416 |
| **Critical commercial assays** | | |
| Annexin A5-FITC kit | Immunotech BeckmanCoulter, Marseille, France | IM3546,  RRID:AB_130885 |
| LEGENDplex™ Mouse HSC Myeloid Panel (7-plex) with Filter Plate | BioLegend, San Diego, CA, USA | 740682 |
| **Softwares** | | |
| FlowJo_v10.8.1 | Tree Star Inc. Ashland, OR, USA | <https://www.flowjo.com/solutions/flowjo>, RRID:SCR_008520 |
| GraphPad Prism version 6.07 | GraphPad Software, La Jolla, CA, USA | [www.graphpad.com](http://www.graphpad.com)  RRID:SCR_002798 |
| ZEN software, blue edition | Carl Zeiss AG, Oberkochen, Germany | <https://www.zeiss.com/microscopy/en/products/software/zeiss-zen.html>, RRID:SCR_013672 |
| R | Bioconductor | [www.r-project.org/](http://www.r-project.org/) RRID:SCR_001905 |
| LEGENDplex™ Data Analysis Software | BioLegend, San Diego, CA, USA | <https://www.biolegend.com/en-us/immunoassays/legendplex/support/software> |
